# Position selectivity in face‐sensitive visual cortex to facial and nonfacial stimuli: an fMRI study†

**DOI:** 10.1002/brb3.542

**Published:** 2016-09-04

**Authors:** David F. Nichols, Lisa R. Betts, Hugh R. Wilson

**Affiliations:** ^1^ Department of PsychologyRoanoke CollegeSalemVAUSA; ^2^ Centre for Vision ResearchYork UniversityTorontoONCanada

**Keywords:** contralateral, fMRI, support vector machine, visual cortex, visual fields

## Abstract

**Background:**

Evidence for position sensitivity in object‐selective visual areas has been building. On one hand, most of the relevant studies have utilized stimuli for which the areas are optimally selective and examine small sections of cortex. On the other hand, visual field maps established with nonspecific stimuli have been found in increasingly large areas of visual cortex, though generally not in areas primarily responsive to faces.

**Methods:**

fMRI was used to study the position sensitivity of the occipital face area (OFA) and the fusiform face area (FFA) to both standard rotating wedge retinotopic mapping stimuli and quadrant presentations of synthetic facial stimuli. Analysis methods utilized were both typical, that is, mean univariate BOLD signals and multivoxel pattern analysis (MVPA), and novel, that is, distribution of voxels to pattern classifiers and use of responses to nonfacial retinotopic mapping stimuli to classify responses to facial stimuli.

**Results:**

Polar angle sensitivity was exhibited to standard retinotopic mapping stimuli with a stronger contralateral bias in OFA than in FFA, a stronger bias toward the vertical meridian in FFA than in OFA, and a bias across both areas toward the inferior visual field. Contralateral hemispheric lateralization of both areas was again shown using synthetic face stimuli based on univariate BOLD signals, MVPA, and the biased contribution of voxels toward multivariate classifiers discriminating the contralateral visual field. Classifiers based on polar angle responsivity were used to classify the patterns of activation above chance levels to face stimuli in the OFA but not in the FFA.

**Conclusions:**

Both the OFA and FFA exhibit quadrant sensitivity to face stimuli, though the OFA exhibits greater position responsivity across stimuli than the FFA and includes overlap in the response pattern to the disparate stimulus types. Such biases are consistent with varying position sensitivity along different surfaces of occipito‐temporal cortex.

## 1 Introduction

Early theoretically driven descriptions of the distribution of function within the visual cortex proposed a progression from initially highly position sensitive processing to later position insensitivity but with increasingly specialized processing, for example, object‐selective cortex (Mishkin, Ungerleider, & Macko, 1983). Psychophysical adaptation studies have indicated both position invariance and position sensitivity for faces (see Zimmer & Kovács, 2011; for review) leaving it unclear as to where in the visual cortex position sensitivity may end. Position sensitivity has now clearly been demonstrated in face‐sensitive cortex using fMRI – initially with a foveal bias (Levy, Hasson, Avidan, Hendler, & Malach, 2001; Hasson, Levy, Behrmann, Hendler, & Malach, 2003; but see Yue, Cassidy, Devaney, Holt, & Tootell, 2011), then a contralateral visual field bias (Hemond, Kanwisher, & Op de Beeck, 2007), then sensitivity to position along the vertical meridian (Schwarzlose, Swisher, Dang, & Kanwisher, 2008), and finally quadrant specificity (Kravitz, Kriegeskorte, & Baker, 2010). Such sensitivity follows logically from restricted spatial receptive fields that show limited position tolerance, as had previously been shown by object‐selective IT neurons in monkeys using electrophysiology (see DiCarlo & Maunsell, 2003) and more recently in face‐sensitive neural patches in monkeys using fMRI (Rajimehr, Bilenko, Vanduffel, & Tootell, 2014), but it was still necessary to show similar effects in humans. There appears to be an increasing trend toward position tolerance in object‐selective areas from the posterior‐lateral areas, for example, Occipital Face Area (OFA), to the more ventro‐medial areas, for example, Fusiform Face Area (FFA) (Cichy et al., 2013; Kovács, Cziraki, Vidnyánszky, Schweinberger, & Greenlee, 2008; Schwarzlose et al., 2008; Taylor & Downing, 2011). For instance, in OFA the response to a stimulus in the ipsilateral visual field is only around 50% of the response to the same stimulus presented in the contralateral visual field, whereas in FFA the ipsilateral response is 75% of the contralateral response (Hemond et al., 2007). Position sensitivity, therefore, indicates either a utility in retaining position information in learning about and acting on objects (DiCarlo & Cox, 2007) or perhaps as a necessary consequence of limited‐size spatial receptive fields (Kravitz, Vinson, & Baker, 2008). Whether positional information is beneficially incorporated in the response profile of object‐selective areas or merely residual response properties from feedforward activation across visual cortex may hinge on the nature of the information that drives neuronal activation within an area. Object‐specific activation would support the former, whereas retinotopic activation would support the latter.

Fusiform face area clearly responds more to faces than other objects (Kanwisher, McDermott, & Chun, 1997; see Kanwisher & Yovel, 2006; for review), as does OFA (Gauthier et al., 2000; see Pitcher, Walsh, & Duchaine, 2011; for review). However, they do not respond exclusively to stimuli composed of entire faces as both FFA (Wilkinson et al., 2000) and OFA (Betts & Wilson, 2010) respond to concentric circles. Plus, synthetic faces (Loffler, Yourganov, Wilkinson, & Wilson, 2005) and line drawings (Kravitz et al., 2010) clearly show that geometric information is sufficient to drive FFA. What remains unclear is to what extent these areas respond in a systematic fashion to nonfacial stimuli, perhaps retaining weak retinotopy that is at times relevant for facial processing (Henriksson, Mur, & Kriegeskorte, 2015).

While retinotopic maps were found near the calcarine fissure in humans with some of the earliest imaging techniques (see Wandell & Winawer, 2011; for review), only more recently have retinotopic maps been shown in a greater range of visual regions anterior to V3 (Brewer, Liu, Wade, & Wandell, 2005). Along the ventral stream, retinotopic maps have been found to overlap with early object‐selective cortex (see Grill‐Spector & Weiner, 2014; for review). However, the face‐selective regions do not overlap with the retinotopic regions (Grill‐Spector & Weiner, 2014; Halgren et al., 1999; Wandell & Winawer, 2011), with face‐selective areas lateral to hV4 and VO2 (Brewer et al., 2005). It is possible that remaining position sensitivity in these areas is due to connections between areas, including early visual cortex (Kravitz, Saleem, Baker, Ungerleider, & Mishkin, 2013; Op de Beeck, Haushofer, & Kanwisher, 2008). If such connections are driving weak position signals then they may easily be overridden by stronger object‐selective signals with more position tolerance. While the type of stimuli used to evidence the retinotopic maps might influence how clearly they can be seen (Alvarez, de Haas, Clark, Res, & Schwarzkopf, 2015), with object stimuli revealing maps better than standard checkerboard patterns in higher visual areas (Henriksson, Karvonen, Salminen‐Vaparanta, Railo, & Vanni, 2012) and biases in the positional representation of nonfacial stimuli (Silson, Chan, Reynolds, Kravitz, & Baker, 2015), there is still no evidence for a clearly organized spatial retinotopic map in OFA or FFA.

The lack of clearly defined retinotopic maps does not preclude position sensitivity based purely on localized receptive fields. Potential responses to position with nonfacial stimuli, that is, retinotopic localizers, will first be explored with the approach that weak signals may not reach standard statistically significant thresholds in order to be revealed in standard maps but may still exhibit biased distributions. Then this study will re‐examine the quadrant position sensitivity previously shown in FFA (Kravitz et al., 2010) and verify that it is similarly present in OFA using typical univariate BOLD and multivariate pattern classification methods, and additionally using a recently developed analysis that looks at the relative contribution of different areas to particular classifiers (Nichols, Betts, & Wilson, 2010). Lastly, we will use a novel analysis to see if any potential retinotopic information corresponds to positional information used to classify the location of face stimuli. Given that location information has been found to translate across category (Cichy et al., 2013), with less position sensitivity in FFA than OFA (Cichy et al., 2013; Schwarzlose et al., 2008), we expect that the nonfacial retinotopic classifiers will be better at classifying the position of face stimuli in OFA than FFA. While much of the results are expected to replicate earlier findings of positional sensitivity with facial stimuli in face‐sensitive visual cortex, the novel use of standard, retinotopy stimuli will allow for a more in‐depth study of the positional sensitivities within these areas.

## 2 Methods

### 2.1 Subjects

Eight subjects (all right‐handed; three females) participated in all experiments, including two of the authors. Seven additional subjects were included in the analysis of responses to nonfacial stimuli as they had previously participated in other experiments where both retinotopic mapping and functional localizers of the OFA and FFA were conducted (Nichols et al., 2010). Subjects were all healthy, paid volunteers, and ranged in age from 21 to 36 years old. Demographic information for all subjects is presented in Table 1. Informed consent was obtained and all procedures were approved by the Research Ethics boards of York University and St. Joseph's Healthcare Hamilton.

**Table 1:** Demographic information for each subject, including age and gender, as well as the type of stimulus viewed and the number of voxels in each of the different regions of interest

Subject	Age	Gender	Stimuli viewed	Retinotopy stimuli	V1	OFA LH	OFA RH	FFA LH	FFA RH
1	31/34	F	Both	Abstract	1400	51		69	69
2	29/31	M	Both	Abstract	1379	6	142	85	98
3	31	M	Both	Simple	1294	177	101	114	109
4	30	M	Both	Simple	1427	127	159	12	79
5	25	M	Both	Simple	902	50	172	26	91
6	23	F	Both	Simple		25	35	32	43
7	22	F	Both	Simple	1177	5	42	41	88
8	21	M	Both	Simple	1208	77	35	30	62
9	31	F	Nonfacial	Abstract	886	47	41	56	112
10	29	F	Nonfacial	Abstract	660	60	68	36	89
11	36	M	Nonfacial	Abstract	1314	55	137	84	117
12	23	M	Nonfacial	Abstract	912	139	151	90	59
13	24	F	Nonfacial	Abstract	982	3	77	9	102
14	23	M	Nonfacial	Simple	1296	28	34	107	188
15	24	M	Nonfacial	Simple	1038	52	93	91	105
$\overset{¯}{x}=$					1133.9	60.1	91.9	58.8	94.1

FFA, fusiform face area; OFA, occipital face area.

A minimum of ten voxels was required in a particular region of interest for inclusion of the data for a subject in a particular analysis.

### 2.2 fMRI data acquisition

Data were acquired on a research 3T short bore GE Excite‐HD magnet equipped with a customized 8‐channel head coil at the Imaging Research Centre, St. Joseph's Hospital, Hamilton, Ontario, Canada. Functional 2D images (T2* weighted gradient echo (EPI), axial plane) consisted of 18–22 slices (4.0 mm thick) that extended from the top of the corpus callosum to the bottom of the temporal lobe (3.75 × 3.75 mm, 35 ms TE, 1250 ms TR, 90° FA, 24 cm FOV, interleaved acquisition, zero gap). Functional images were aligned to a high‐resolution SPGR whole‐brain anatomical scan (0.5 × 0.5 × 0.8 mm, FastIR prep, Zip512, T1 weighted, 12° FA, 24 cm FOV, 2.1 ms TE). Data were first processed in Brain Voyager QX (v 1.10) and then analyzed in Matlab (v 7.4 R2007a).

### 2.3 Retinotopic localizers

**Figure 1:**
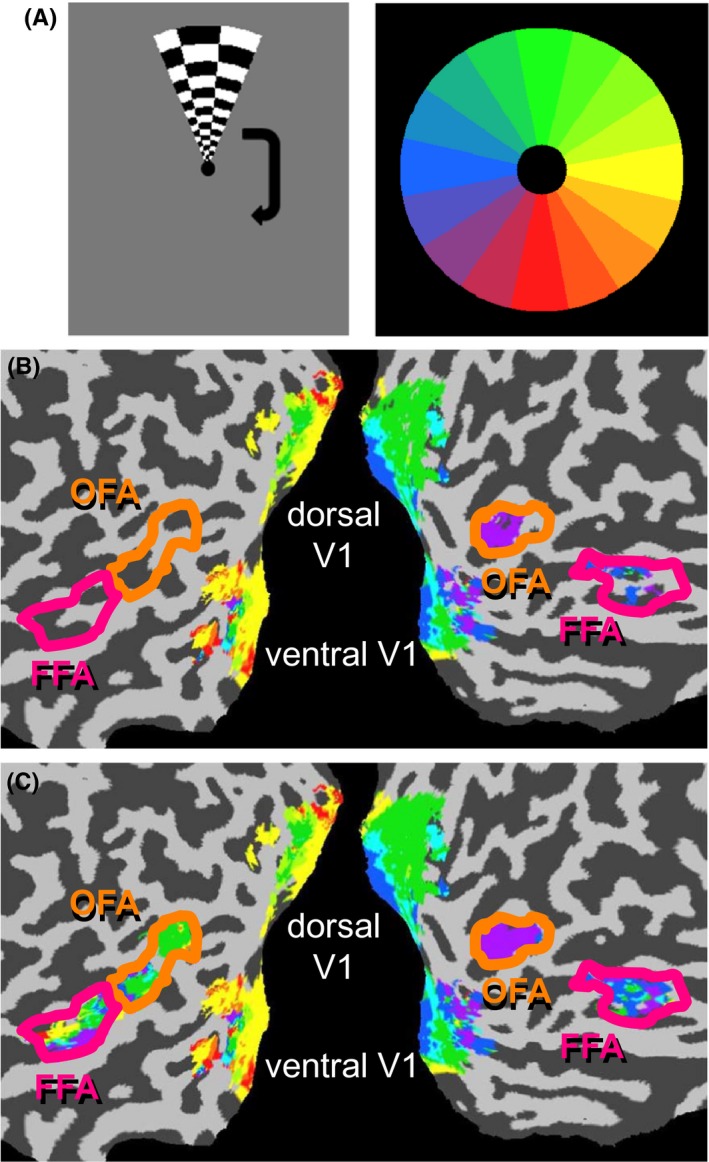
Retinotopic mapping results within regions of interest for a single representative subject. (A) Rotating wedges containing rapidly changing stimuli were shown at eight locations around an imaginary circle. Polar angle lag values could take on one of sixteen different values depicted in the color wheel. (B) Lag value maps for V1, occipital face area (OFA), and fusiform face area (FFA) when applying a threshold for significant correlation (*p *<* *.001). (C) Lag value maps for V1, OFA, and FFA when no threshold for inclusion was applied.

All subjects viewed one of two types of polar angle and eccentricity retinotopic localizer scans, generally presented at the end of the session. Two participants viewed the localizer scans and the quadrant facial stimuli scans a few years apart. All localizer scans followed standard localizer procedures (Engel, Glover, & Wandell, 1997; Sereno, McDonald, & Allman, 1994). For the polar angle localizer, the position of a wedge‐shape region, 9° high and 3° wide at 4° eccentricity, moved in eight discrete steps separated by 45° (i.e., nonoverlapping, adjacent positions) around an imaginary circle eight times (see Fig. 1A). For the eccentricity localizer, an annular region 0.5° thick moved in eight discrete steps of eccentricity (nonoverlapping, adjacent positions, except for an abrupt change from the outer to the inner position at the end of each cycle) a total of eight times. Each step was presented for 8 s, with constant updating of the image within the stimulus region. A lag value was assigned to each voxel, which was the phase of the rotating wedge that resulted in the greatest amount of activation. This lag value was taken as the portion of the visual field that a particular voxel responded to the most. The 16 possible lag values were referenced with regards to a clockwise rotation, with 1 as the 12 o'clock position, 5 as the 3 o'clock position, 9 as the 6 o'clock position, and 13 as the 9 o'clock position.

For eight of the 15 subjects, the stimulus regions were filled with drifting, expanding/contracting, and counterphasing checkboard patterns of varying check sizes. These are referred to in Table 1 as “simple” retinotopy stimuli. For the other seven subjects, the stimulus regions were filled with drifting complex abstract art images of a variety of different paintings, with a small subset containing face images. These are referred to in Table 1 as “abstract” retinotopy stimuli. The purpose of using abstract art images was to present interesting, engaging stimuli that still contained a variety of low‐level image features. However, there were no clear biases in the number of voxels included in the ROIs for the different types of localizer stimuli and the results were consistent regardless of the type of localizers used. Therefore, results are presented with the data of all subjects analyzed together.

### 2.4 Facial stimuli presentation

Stimuli were presented in 15 s blocks at one position at a time, with 15 s fixation blocks between each experimental block. Individual stimuli were presented for 2 s, with 0.5 s in between. Three blocks of each position were viewed in each of 5 scans, resulting in a total of 15 blocks per position. The stimuli were offset from the central fixation point by an average of ±3° vertically and ±2° horizontally. This resulted in four distinct positions presented – superior left (SL), superior right (SR), inferior left (IL), and inferior right (IR). Each image was jittered by no more than 0.5° vertically and horizontally from the previous image in order to avoid local adaptation during a block. Stimuli were the whole face, internal features, or external head outlines of synthetic face stimuli, constructed from a database of 80 grayscale photographs (Betts & Wilson, 2010; Wilson, Loffler, & Wilkinson, 2002). (For the purposes of this study, differences between the responses to the different types of facial stimuli were not analyzed, as this was the subject of Nichols et al. (2010)). The average size of the whole face stimuli was 5° high by 3° wide. The percent signal change was averaged across the final 11.25 s of a 15 s block of stimuli in order to determine the response for each position. Examples of the stimuli and their relative offset can be seen in Fig. 3A.

Subjects performed a demanding 1‐of‐3 fixation color detection task. At random intervals, independent of the presence or absence of a stimulus or stimulus block, the color of the central portion of a dark gray fixation dot was red or green or blue or dark gray (i.e., uniform). Subjects were required to press a different button, depending on which nongray color the fixation dot was. The maintenance of fixation was not explicitly verified using eye‐tracking devices, but if the stimuli were fixated directly or eye movements were random and uniformly distributed, classification of the stimulus position would not have been possible in retinotopic cortex, though it clearly was. This indicates that subjects maintained fixation in the vicinity of the fixation dot.

### 2.5 Functional localizer

Interspersed among the experimental scans were two functional localizer scans designed to isolate face‐sensitive regions of human visual cortex. The scans included 16 s blocks of face or house stimuli, presented in random order, with stimulus blocks always separated by 30 s fixation blocks. The face stimuli were grayscale photographs of front view and side view faces, cropped closely to the outline of the head, and averaged 8° high by 5° wide. The house stimuli were grayscale photographs of front view and side view houses, cropped to fit in 9° squares, which included portions of the surrounding yard and sky. These stimuli have been used for functional localizers for previous studies in this laboratory (e.g., Loffler et al., 2005). Six pairs of stimuli, which all included a front view and side view image, were presented per block, with each pair lasting 1.5 s and separated by 0.5 s of just a fixation dot between pairs. Subjects were instructed to push a button whenever the pair was of the same face or house.

### 2.6 Definition of regions of interest

A general linear model (Brain Voyager QX, V. 1.10) was applied individually to each subject's data in native, that is, non‐Talairach, brain space. The Bonferroni‐corrected contrast between activation to Face and House blocks as well as anatomical markers were used to define the FFA and OFA regions of interest. A Talairach transformation applied to the native Brain Voyager coordinates confirmed that the identified regions of interest corresponded well to previously reported locations of face‐sensitive visual cortex (Mean coordinates for 15 observers: OFA_LH_ [−37, −74, −9]; OFA_RH_ [41, −71, −9]; FFA_LH_ [−36, −50, −17]; FFA_RH_ [38, −50, −15]).

### 2.7 Multivoxel pattern analysis methods

A linear, multiclass support vector machine (SVM) classifier using SVMKMToolbox (Canu, Grandvalet, Guigue, & Rakotomamonjy, 2005) was established using the procedure detailed in Kamitani and Tong (2005). The activation vectors for one scan were left out to be used in the testing phase, whereas the remaining samples across the other scans were used for training. A classifier for each category, which consisted of one of the four quadrant stimulus positions shown in Fig. 3A, was created by first establishing the three pair‐wise classifiers for each category with the other three categories, then summing together the output of the SVM procedure, that is, the weight vectors and the biases, of each pair‐wise classifier (see Nichols et al. (2010) for more details). (For the purposes of this study, position classifiers were defined after collapsing across the different types of facial stimuli, as classifying different types of facial stimuli was the subject of Nichols et al. (2010)). Each of the left‐out samples was then classified based on which of the four category classifiers resulted in the largest positive output. Then a different scan was left out for testing and the procedure was repeated using the new set of training samples until each sample of each category was used exactly once as a test. Then the proportion of samples that were correctly classified was determined.

In order to determine the relative contribution of each hemisphere within OFA or FFA to the classification of the quadrant position of the facial stimuli, the relative distribution of voxels contributing the most to each of the four positions was determined (see Nichols et al. (2010), for justification and details on this method). In short, each voxel was assigned to a single category based on which of the four quadrant position categories it contributed the strongest positive weighting for. The relative frequency of each category within each ROI was calculated separately for each participant and the patterns across ROIs were analyzed for consistency across participants. Classification was done with both hemispheres contributing inputs to the classifiers, but voxels from OFA and FFA contributed to separate classifiers. Voxels from the right and left hemispheres of V1 were also analyzed in a similar way to demonstrate the output of the method on an area with a very well‐established contralateral bias.

### 2.8 Creation of retinotopy‐based position classifiers

In the earlier analysis of the retinotopic mapping within OFA and FFA, each voxel was assigned a particular lag value (see 2.3 section above for details). Classifiers for the presentation of a stimulus within each of the four quadrants of visual field were constructed through differential weighting of the voxels depending on their assigned lag values. For a given quadrant, for example, superior left (SL), all voxels with a lag value within that quadrant, for example, 14, 15, and 16, were given a strong positive weight, +2, as their response was highly consistent with a stimulus in that quadrant. Voxels with lag values consistent with nearby quadrants or meridian values, for example, 1–4 and 10–13, were given a weaker positive weight, +1, as their response is somewhat consistent with a stimulus in that quadrant. All remaining voxels with lag values consistent with the opposite quadrant, for example, 5–9, were given a negative weight, −1, as their response was inconsistent with a stimulus in that quadrant. A classifier of this type was created for each quadrant. Then, each weight vector for the different position classifiers was normalized to have a mean of 0 and root mean square of 1 to balance out the magnitude of responses for the different position categories due to different relative numbers of voxels for the various preferred polar angles. For each trial, the BOLD response across all voxels was combined with each of the four classifiers and the output of the trial was taken as the quadrant classifier with the highest response.

As the true position of the stimulus was known to the researcher, that is, whether it was truly presented in the left (L) or right (R) and inferior (I) or superior (S) visual field, each trial could be scored regarding the location of the output in relation to the true quadrant. Collapsing across all quadrants, each trial was scored as being the correct quadrant (correct L|R and correct I|S), the incorrect quadrant but correct hemifield (correct L|R, incorrect I|S), etc. If there is a sufficiently high number of receptive fields within face‐sensitive cortex that are driven by both nonfacial and facial stimuli due primarily to their relative position within the visual field, then it ought to be possible to classify the quadrant correctly at above chance levels, that is, greater than 25% correct. Furthermore, if a contralateral hemifield bias exists within different ROIs and the receptive fields are not too highly selective to stimulus type, incorrect outputs ought to be biased toward the correct L|R visual field.

## 3 Results

### 3.1 Analysis of pure retinotopy using nonfacial stimuli

Preferred polar angle was determined on a voxel‐by‐voxel basis by a correlation analysis on responses to standard nonfacial retinotopic mapping stimuli, identifying the highest correlation between a theoretical hemodynamic response function with temporally offset versions of the time course to the repeating rotational stimulus shown in Fig. 1A. Although standard retinotopic mapping generally applies a threshold to the correlation values prior to plotting the preferred polar angle in brain maps, as in Fig. 1B, the preferred polar angle for all voxels, regardless of correlation strength, was utilized for further analysis, as shown in Fig. 1C. This was done based on the assumption that weak positional biases that would otherwise be missed when a threshold was applied may be revealed through population statistics when all voxels are included in the analysis.

**Figure 2:**
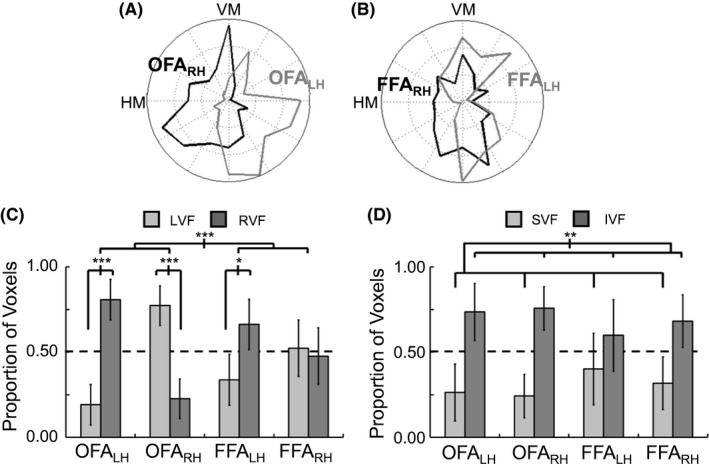
Biases in the lag values for occipital face area (OFA) and fusiform face area (FFA) to nonfacial stimuli. (A) and (B) Distribution of greatest polar angle sensitivity across voxels in the OFA and FFA, respectively, grouped based on left hemisphere (LH) and right hemisphere (RH). (C) Analysis of horizontal visual field bias (left vs. right visual field) based on the relative distribution of lag values across voxels, excluding those with preferred lag values along the vertical meridian (VM). (D) Analysis of vertical visual field bias (superior vs. inferior visual field) based on the relative distribution of lag values across voxels, excluding those with preferred lag values along the horizontal meridian (HM). (error bars = 95% c.i.) (*p <* *.05, **p <* *.01, ***p <* *.001)

The patterns of preferred polar angles for left and right hemisphere, averaged across subjects, are shown separately for the OFA and FFA (Fig. 2A and B). From these plots, clear differences can be observed between the OFA and FFA, with the OFA showing strong lateralization of hemispheres biased toward the contralateral visual field, but with both hemispheres of the FFA seeming to cluster near the vertical meridians, without as much lateralization. Furthermore, all ROIs show a bias toward the inferior visual field. The reliability of these observations across subjects was tested by grouping the preferred polar angles either based on left‐right visual field, inferior‐superior visual field, or proximity to the vertical‐horizontal meridian prior to statistical analysis.

For each subject, the percentage of voxels that fell within each visual field or in the vicinity of the meridians was calculated, with individual voxels allowed to count toward more than one analysis. For the left‐right visual field bias, the proportion of voxels with preferred polar angles in the right visual field in relation to those in the left visual field, excluding those directly on the vertical meridian, was calculated within each ROI. For the inferior‐superior visual field bias, a similar calculation was made with the proportion of voxels with preferred polar angles in the superior visual field in relation to those in the inferior visual field, excluding those directly on the horizontal meridian, calculated within each ROI. For the vertical‐horizontal meridian bias, the proportion of voxels was calculated with preferred polar angles in the vicinity of the vertical meridian, including those directly on and one lag before and after the vertical meridian, in relation to those directly on and one lag before and after the horizontal meridian.

Prior to running statistics, individual ROIs were excluded for subjects that did not have at least 10 voxels within that ROI (see Table 1). As a result, data for only 10 of 15 subjects was included in all ROIs. In order to test for consistent biases across subjects, a 2 × 2 repeated measures MANOVA was run as there are clear correlations between the dependent variables, with area (OFA and FFA) and hemisphere (right and left) as factors. Regarding the overall MANOVA, the main effect of area was not significant (*F*_3,7_ = 2.70, *p *=* *.126) but there was a main effect of hemisphere (*F*_3,7_ = 11.08, *p *=* *.005) and an interaction effect of area and hemisphere (*F*_3,7_ = 15.25, *p *=* *.002). There were clear differences in the pattern of effects across dependent variables.

With respect to left‐right visual field lateralization (see Fig. 2C), a significant area by hemisphere interaction (*F*_1,9_ = 27.24, *p *<* *.001) and main effect of hemisphere (*F*_1,9_ = 27.78, *p *<* *.001) was present. Further tests confirmed that the lateralization effect was stronger in the OFA than FFA. Within OFA, both hemispheres exhibited a strong contralateral bias (RH = 77%, LH = 81%, all *p*‐values <.001), but within the FFA, the left hemisphere exhibited a bias (66%, *p *=* *.040), whereas the right hemisphere did not (52%, *p *=* *.770). However, paired t‐tests between the proportion of voxels preferring the right visual field, conducted separately in the OFA and FFA, demonstrated that lateralization was significant in both the OFA (*t*_10_ = 9.87, *p *<* *.001) and FFA (*t*_13_ = 2.40, *p *=* *.032).

With respect to inferior‐superior visual field bias (see Fig. 2D), neither the main effects nor the interaction effect were significant (all *F*‐values < 1.5, all *p*‐values > .25). However, there was a consistent bias across all ROIs for a higher proportion of voxels preferring polar angles in the inferior visual field. This was determined by averaging across all ROIs to establish a single measurement of the bias for each subject. Then a single‐sample *t*‐test was run against a value of 0.5, with the null‐hypothesis of a lack of bias. The resulting statistic (*t*_14_ = 3.63, *p *=* *.003) confirmed the inferior visual field bias (68%).

With respect to a vertical‐horizontal meridian bias, a main effect was found for area (*F*_1,9_ = 6.39, *p *=* *.032) but there was no main effect of hemisphere nor an interaction effect (all *F*‐values << 1). Therefore, the presence or absence of a bias was tested separately within each area after averaging across hemisphere. A significant bias toward the vertical meridian was found in the FFA (72%, *t*_14_ = 3.92, *p *=* *.002) but not in the OFA (57%, *t*_14_ = 1.19, *p *=* *.250).

### 3.2 Position sensitivity using quadrant presentations of facial stimuli

**Figure 3:**
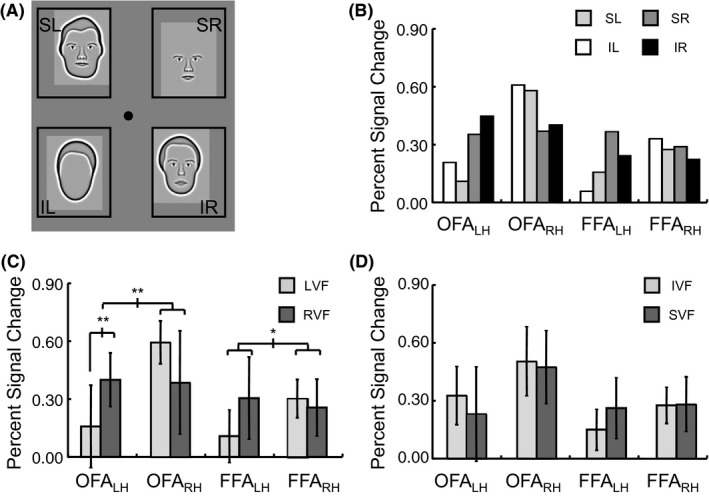
Facial stimuli and response amplitude to different positions. (A) Example stimuli shown at the four quadrant locations, though a stimulus in only a single quadrant was shown within each block. Analysis was done after collapsing across responses to different types of facial stimuli. (B) Activation to each of the four positions in face‐sensitive regions of interest. (C) Analysis of horizontal visual field bias (SL/IL for LVF vs. SR/IR for RVF) based on the average response within an entire region of interest. (D) Analysis of vertical visual field bias (SL/SR for SVF vs. IL/IR for IVF) based on the average response within an entire region of interest. (error bars = 95% c.i.) (*p <* *.05, **p <* *.01, ***p <* *.001)

Position sensitivity to facial stimuli as evidenced by activation levels averaged across all voxels within an ROI was determined by measuring the BOLD response level within each hemisphere of the OFA and FFA to four different positions of facial stimuli (Fig. 3A). The response level per position within each ROI is shown in Fig. 3B. To separately assess the reliability across subjects of the lateralization of visual field between hemispheres, statistics were performed after collapsing across either the two vertical positions or the two horizontal positions. With respect to a left‐right visual field lateralization, which would be consistent with a contralateral visual field bias, a significant visual field by hemisphere by ROI interaction (*F*_1,3_ = 20.31, *p *=* *.020) was found when a Repeated Measures ANOVA was performed, indicating that the lateralization was stronger in the OFA than FFA (Fig. 3C). However, when the ROIs were separately analyzed, a strong interaction between visual field and hemisphere was found in both the OFA (*F*_1,4_ = 36.20, *p *=* *.004) and FFA (*F*_1,6_ = 11.63, *p *=* *.014). When each hemisphere within each ROI was individually analyzed regarding the response to the contralateral and ipsilateral visual field, paired t‐tests showed that the left hemisphere OFA showed a significant difference (*t*_6_ = 3.98, *p *=* *.007) and the right hemisphere OFA was marginally significant (*t*_5_ = 2.12, *p *=* *.087), but neither hemisphere in FFA showed a consistent difference (all *t*‐values < 1.8, all *p*‐values > .10).

With respect to the superior‐inferior visual field bias, neither the repeated measures main effect for visual field (*F *<< 1) nor interactions involving visual field (all *F*‐values < 2.9, all *p*‐values > .18) were significant (Fig. 3D). While this may be somewhat surprising, given that an inferior visual field bias in FFA has previously been demonstrated (Schwarzlose et al., 2008), large differences in the type and position of the stimuli used in the respective studies may have contributed to whether or not the effect was found. Perhaps most importantly, their stimuli were presented along the vertical meridian, whereas we presented the stimuli away from the vertical meridian, within separate visual field quadrants. More recently a superior visual field bias was found in right FFA that was distinct from an inferior visual field bias in right OFA using scene stimuli (Silson et al., 2015). Our data showed a slight but insignificant pattern in that direction across both hemispheres of OFA and FFA. However, note that we found an inferior visual field bias for the nonfacial retinotopic stimuli that included stimuli on and near the vertical meridian, so large scale differences in the stimuli between the studies could explain the disparate findings.

**Figure 4:**
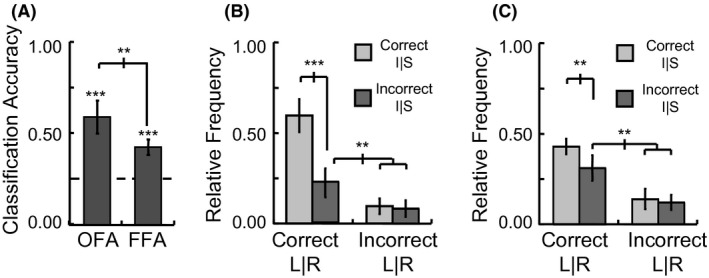
Results of multivoxel pattern analysis within each face‐sensitive region of interest, with voxels combined across hemispheres. (A) The proportion of trials that were correctly classified according to quadrant in occipital face area (OFA) and fusiform face area (FFA). (B) and (C) Distribution of outputs of the SVM multiclass classifiers in the OFA and FFA, respectively, including incorrect trials. I and S refer to the inferior and superior visual field, respectively, and L and R refer to the left and right visual field, respectively. (error bars = 95% c.i.) (*p <* *.05, **p <* *.01, ***p <* *.001)

The nature of patterns of activation across hemispheres to different spatial positions within entire areas was analyzed using multivoxel pattern analysis (Kamitani & Tong, 2005). As facial stimuli were presented in one of four visual quadrants throughout an entire block and all four quadrants were presented an equal number of times within each of five scans, the blocks from one scan were left out for testing, whereas the blocks from the other four scans were used as samples for training. The voxels from the right and left hemisphere of an area were combined prior to classification, which was necessary given the observed contralateral biases in both OFA and FFA (Fig. 3C). Classification of the position of the stimuli (Fig. 4A) was at above chance levels (i.e., >25%) in both the OFA (60% percent correct, *t*_7_ = 8.97, *p *<* *.001) and FFA (43% percent correct, *t*_7_ = 9.94, *p *<* *.001). A paired t‐test indicated that classification performance of position was reliably higher in OFA than FFA (*t*_7_ = 3.51, *p *=* *.010).

Additional positional sensitivity can be determined within areas by examining trials for which classification was not correct (Fig. 4B and C). Both OFA and FFA showed errors that were systematically biased toward the same left‐right visual field as the correct position (55% of trials, with chance at 33%, *t*_7_ > 3.5, *p *<* *.01 in both areas), consistent with a contralateral visual field bias. Evidence for differentiation between the vertical positions of the stimuli in addition to the horizontal differentiation was shown through paired t‐tests for the guesses that were in the correct horizontal position. The output of the classification procedure was more frequently in the correct than the incorrect vertical position for both the OFA (*t*_7_ = 5.50, *p *=* *.001) and FFA (*t*_7_ = 3.81, *p *=* *.007). This ability to distinguish the vertical position as well as the horizontal position of a face indicates quadrant sensitivity in both the OFA and FFA.

**Figure 5:**
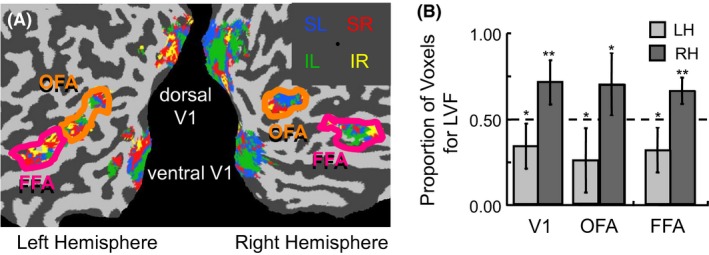
Results of the spatial distribution of the multivoxel pattern classifiers across the four stimulus positions. (A) Voxel category maps a single representative subject with color‐coded voxels categorized based on which position classifier they contributed the strongest supportive weighting to, separated by region of interest. Noisy categorization based on noninformative voxels contributes to decreased representation of spatial processing within the maps, as can be seen in the distribution of quadrant coloring within V1. (B) Analysis of horizontal visual field bias (SL/IL vs. SR/IR) based on the relative frequency of voxel categorization across position classifiers. (error bars = 95% c.i.) (*p <* *.05, **p <* *.01, ***p <* *.001)

Thus far our analyses regarding positional sensitivity to facial stimuli have been done combining across all voxels within an ROI. Replication of contralateral bias at the voxel level was assessed by ascertaining the spatial distribution of which position classifier the voxels contributed most strongly to (Fig. 5A; see 2 and Nichols et al. (2010) for details on how this was done). As shown in Fig. 5B, it was found that a higher proportion of voxels in the right hemisphere contributed most strongly to the classifiers of left visual field positions in both the OFA (*t*_4_ = 3.05, *p *=* *.038) and FFA (*t*_6_ = 4.93, *p *=* *.003), whereas the left hemisphere voxels showed a bias toward contributing most strongly to the classifiers of right visual field positions in both the OFA (*t*_4_ = 3.61, *p *=* *.023) and FFA (*t*_6_ = 3.34, *p *=* *.016). For confirmation of the methodology in an area with well‐established contralateral field hemispheric lateralization (e.g., Wandell & Winawer, 2011), V1 was tested with the same analysis and similar results were found (RH: *t*_6_ = 4.00, *p *=* *.007; LH: *t*_6_ = 3.06, *p *=* *.022). A 3 × 2 Repeated Measures ANOVA on the proportion of voxels contributing most strongly to the right visual field classifiers with area (FFA, OFA, V1) and hemisphere (RH, LH) as factors showed a significant main effect of hemisphere (*F*_1,3_ = 14.61, *p *=* *.032) but not of area (*F* < 1) nor was there an interaction effect (*F*_2,6_ = 1.94, *p *=* *.223). Note that although direct interpretation of weight vectors as an absolute measure of a voxel's contribution to encoding a particular neural representation would be a flawed practice (Haufe et al., 2014), we are using it as a relative measure of bias so a high degree of incorrect voxel assignments actually contributes noise to the data that works against finding our observed biases. Though it would be imprudent to claim a similar level of contralateral bias across all areas, such a pattern of results is consistent with the voxel classification method being a valid demonstration of a contralateral bias within each area.

### 3.3 Test for contribution of pure retinotopy in position sensitivity for facial stimuli

**Figure 6:**
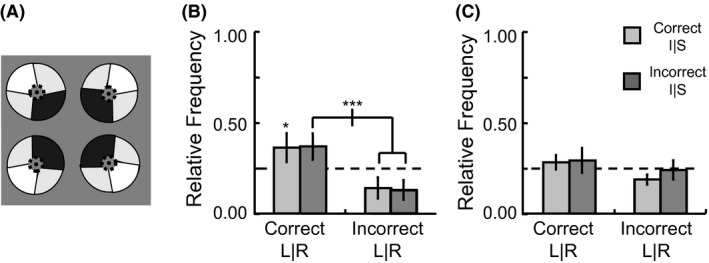
Results of the classification of the position of facial stimuli based on four quadrant position classifiers generated from nonfacial stimuli. (A) The relative weighting of voxels with different polar angle lag values to each of the four position classifiers, with white showing a strong positive weight, light gray a weak positive weight, and black a weak negative weight. The position of the four annuli within the figure indicates the position they respond to the most. When applied to the activation vector across voxels to each trial, the classifier with the greatest positive value was taken as the position output for that trial. (B) and (C) Distribution of outputs of the position classifiers for the occipital face area (OFA) and fusiform face area (FFA), respectively, for correct and incorrect trials. I and S refer to the inferior and superior visual field, respectively, and L and R refer to the left and right visual field, respectively. (error bars = 95% c.i.) (*p <* *.05, **p <* *.01, ***p <* *.001)

To examine how much of the position‐dependent activation patterns in the OFA and FFA are caused by pure retinotopic responses, a novel generalization procedure was utilized wherein position classifiers were built separately for OFA and FFA based on the nonfacial retinotopic mapping activation patterns and tested on the facial stimuli activation patterns. More specifically, the preferred polar angle lag values were used to determine if the observed spatial preference of individual voxels is reliable and informative regarding response patterns to faces presented at discrete spatial positions (see Fig. 6A and 2 for full details).

Figure 6B and C show the generalization results for the preferred polar angle classifiers in the two ROIs. Classification of which of the four quadrants a facial stimulus was presented in was significantly above chance in the OFA (34% percent correct, *t*_7_ = 2.58, *p *=* *.036), but not in the FFA (28% percent correct, *t*_7_ = 1.86, *p *=* *.105). Further analysis of the trials for which classification was not correct showed that errors were systematically biased toward the same left‐right visual field as the correct position in the OFA (*t*_7_ = 3.33, *p *=* *.013), but not necessarily in the FFA (*t*_7_ = 1.90, *p *=* *.100). These results indicate that the position sensitivity for facial stimuli in the OFA is, at least in part, driven by localized receptive field structure that is general across stimuli, but position sensitivity in the FFA likely requires facial stimuli to be used in order to observe it. However, since FFA showed a stronger vertical meridian bias than OFA, such nonfacial position based stimulus classifiers may work better in FFA for stimuli directly above and below fixation.

## 4 Discussion

Position sensitivity was found for both nonfacial and facial stimuli in both OFA and FFA. Using nonfacial retinotopic stimuli, a contralateral visual field and inferior visual field bias was shown in both areas, whereas an additional bias for the vertical meridian was found in FFA. Using facial stimuli presented in just the four quadrants away from the vertical and horizontal meridians, a contralateral visual field bias was again shown in both areas, though stronger in OFA, but not an inferior or superior visual field bias. The contralateral visual field bias was found in the response levels as well as in the distribution of the voxels that contributed most to the different positional classifiers. Quadrant‐specific positional sensitivity was found in both areas using multivariate classifiers. Evidence for pure positional information driving the response in OFA was shown using classifiers based on each voxel's response to nonfacial retinotopy stimuli to classify visual field position at above chance levels for facial stimuli.

Observed differences between areas may be because OFA processes particular face parts at particular locations in space, whereas FFA processes roughly but not specifically where the entire face is, thus showing greater position tolerance and less transfer from nonfacial, purely retinotopic stimuli. Activation in FFA could indicate that a face is in a particular region with the information flowing back to OFA to process the nature of the particular face part in that particular region, with the general standardized structure of a face providing a strong clue as to where a particular face part, for example, the eyes, might be located in the visual scene (Henriksson et al., 2015). This is in part because FFA is more sensitive to the spatial frequency content of an image over a large spatial range (Rossion, Hanseeuw, & Dricot, 2012). OFA may indicate where different face parts are, with individual face parts being processed largely independently at a variety of spatial locations (Henriksson et al., 2015).

Also consistent with a larger global structure preference in FFA in relation to OFA is that FFA shows a face inversion effect for whole faces but not combinations of face parts, whereas OFA shows differential activation for inverted face parts (James, Arcurio, & Gold, 2013). However, FFA activation also differentiates scrambled faces from scrambled objects (Andrews, Clarke, Pell, & Hartley, 2010; Rossion et al., 2012), indicating selectivity for certain low‐level stimulus information. Interestingly, in the Rossion et al. study, OFA did not show differentiation between scrambled faces and scrambled objects or intact objects, but rather showed a clear preference for intact faces. From this the authors concluded that the FFA is less “face‐selective” than OFA because it responds more to cars than scrambled cars and more to faces than scrambled faces, whereas OFA only responded differentially for faces. However, FFA responded strongly to intact objects and barely above baseline for scrambled objects whereas OFA responded well above baseline to all of the “nonselected” stimuli, indicating perhaps that the low‐level stimulus properties were equal in everything but the intact face stimuli and all better than just fixation. This is consistent with proposals that OFA is less selective to object category than FFA (see Taylor & Downing, 2011, for review).

Also, FFA and OFA showed adaptation only for intact faces in Andrews et al. (2010) indicating a clear difference in processing for faces in relation to other stimuli. That difference may be due to differential sensitivities to particular spatial frequency bands in FFA (Woodhead, Wise, Sereno, & Leech, 2011; Yue, Tjan, & Biederman, 2006) reflecting a preference for whole faces that is not present in the OFA because it is more sensitive to the individual face parts than the global configuration (Liu, Harris, & Kanwisher, 2010). Therefore, even though OFA shows greater position sensitivity, it may not be as concerned with the relative position/configuration of face parts as much as their presence or absence (Liu et al., 2010; Pitcher, Walsh, Yovel, & Duchaine, 2007), that is the stimuli either stimulate or do not stimulate local receptive fields, whereas a matching to larger templates may be required in FFA.

Carrying information about identity preserving transformations throughout the system would eventually become inefficient. An increased specialization to combinations of local features, regardless of their exact position, is a general principle across object‐selective cortex (Wilson and Wilkinson, 2015). DiCarlo and Cox (2007) argue that limited sensitivity to position and pose, for example, within individual neurons in IT cortex may actually aide in identifying objects in the real world. This is because we rarely perceive the same objects under identical viewing conditions and orientations, therefore untangling the aspects that are irrelevant to the object, such as size and position, can leave only those aspects that discriminate between objects. Retaining information about position and size, in particular, also is an efficient means of allowing the visual system access to information such as where a face is and whether it is larger or smaller than average, without having a separate region of cortex dedicated to these properties. There is evidence from MEG that object‐selective cortex retains information about position in early responses but can also respond to object information in a position tolerant way in subsequent processing (Carlson, Hogendoorn, Kanai, Mesik, & Turret, 2011).

Position sensitivity, which can arise from a biased population response with no clear underlying spatial order, is distinct from retinotopy, which specifies an orderly progression in a spatial dimension across cortex, such as polar angle or eccentricity maps found in early visual cortex. Both fMRI in humans (e.g., Grill‐Spector & Weiner, 2014) and fMRI in monkeys (e.g., Rajimehr et al., 2014) have indicated that face‐selectivity begins in regions that border but do not entirely overlap with retintopic visual areas. Given that fMRI voxels appear to provide information at a spatial scale on the order of 3 mm (Issa, Papanastassiou, & DiCarlo, 2013), it is always possible that retinotopy exists at a spatial level that is inaccessible to fMRI. However, perhaps it is more feasible that face‐sensitive regions are organized based on a stimulus‐relevant feature dimension that demonstrates a local spatial bias, such as eye‐region (Issa & DiCarlo, 2012), though is interspersed with processing of spatial parts that have a wide range of components from different locations, such as facial outlines (Nichols et al., 2010). This could then show spatial heterogeneity that would violate retinotopy but still demonstrate population level position sensitivity. A previous neurophysiology study in the posterior monkey face patch also found a contralateral bias, though with a superior visual field bias (Issa & DiCarlo, 2012), distinct from the inferior visual field bias we observed with the nonfacial stimuli. However, another recent neurophysiology study found an inferior visual field bias using face‐based retinotopy stimuli across a wide range of face patches (Rajimehr et al., 2014). Therefore, use of stimuli with different contours, such as spatially extended wedges (e.g., Rajimehr et al., 2014; current study) versus faces (e.g., Issa & DiCarlo, 2012), could contribute to the disparate findings. Such questions may not be able to be fully answered until a single study utilizes spatially extended and spatially localized contours for both facial and nonfacial stimuli.

## 5 Conclusion

Greater spatial biases in positional sensitivities within face‐sensitive areas OFA and FFA were found using nonfacial stimuli compared to using facial stimuli with a contralateral bias being the most consistent finding across analysis techniques. Though this study serves as a replication of positional sensitivity found using facial stimuli presented at different positions within the visual field, new information has been provided in that this study also explored positional sensitivities in these areas using standard retinotopic stimuli and compared results across various analyses. The results overall support the use of low magnitude lag value correlations and distributions of maximally supportive classifier weights to demonstrate biases in the processing across regions of interest. Note that the observed contralateral field bias in voxel distributions does not indicate that the right hemisphere areas selectively process only the left visual field, and vice versa. Position sensitivity across the entire visual field could occur within each hemisphere if there are large receptive fields that are primarily centered within the contralateral visual field, but that are large enough to extend across the midline. Estimates of receptive field sizes for faces from human psychophysics averaged around 10–12° (Afraz & Cavanagh, 2008), large enough to be at least partially stimulated by stimuli across multiple quadrants. Differences between hemispheres in the relative number of receptive fields responding to the vertical midline, particularly the fovea, could influence the observed size of face‐sensitive areas using standard localizers. Results of fMRI studies of faces could change, including which voxels show up in an ROI, if faces at a greater variety of positions are used. Also, a decrease in position sensitivity from the OFA to FFA observed with pure retinotopy stimuli and less generalization of classifiers built based on retinotopy stimuli implies an increase in selectivity as to the nature of the stimuli that can drive the neurons in a particular area. Furthermore, FFA caries more information on perceived position than physical position of stimuli (Fischer, Spotswood, & Whitney, 2011).

Position sensitivity appears to decrease from the OFA to FFA, consistent with the general principal of decreasing spatial sensitivity from posterior to anterior regions of the ventral visual cortex (Schwarzlose et al., 2008). This implies that low‐level stimulus features are no longer carried forward, presumably to allow for more and more specialized processing of particular category relevant information (Wilson and Wilkinson, 2015). Overall it appears that OFA is part of a collection of posterior‐lateral occipito‐temporal cortex areas that are more primitive, local, and stimulus driven in relation to ventro‐medial occipito‐temporal cortex areas, including FFA, that are more global and invariant across visual features (Taylor & Downing, 2011).

## Funding Information

Natural Sciences and Engineering Research Council of Canada (Grant/Award Number: ‘OP227224’) Canadian Institute for Advanced Research, Canadian Institutes of Health Research

## Conflict of Interest

None declared.
